# Supplementary material for: Favorable Humoral Response to Third Dose of BNT162b2 in Patients Undergoing Hemodialysis
Source: J Clin Med. 2022 Apr 8;11(8):2090. doi: 10.3390/jcm11082090 (PMC9024432; doi:10.3390/jcm11082090)
Supplement: Supplementary file 1 [file jcm-11-02090-s001.zip › jcm-1652889-SI.pdf]

SUPPLEMENTARY Table S1. Correlation between continuous parameters and anti-spike immunoglobulin G antibody levels 3 months or 4.5 months after the second dose of BNT162b2.

	Patients on HD (3M)		Healthcare Workers (4.5M)	
	ρ	*p*-Value	ρ	*p*-Value
**Age**	−0.099	0.10	−0.403	<0.001
**Dialysis vintage (months)**	0.144	0.02	NA	
**Body mass index**	−0.075	0.21	0.040	0.64
**White blood cell count**	−0.001	0.98	−0.029	0.74
**Hemoglobin**	−0.065	0.28	−0.076	0.38
**Albumin**	0.105	0.08	NA	
**KT/V**	0.062	0.30	NA	

HD: hemodialysis, NA: not available

Spearman analysis was used

Supplementary Table S2. The effect of sex on anti-spike immunoglobulin G antibody levels at the follow-up of the second dose.

	Male	Female	*p*-Value
**(1) Measurement in October 2021**			
**Patients on hemodialysis (U/mL)**	220 (107–363)	200 (91–408)	0.97
**Healthcare workers (U/mL)**	464 (274–772)	649 (435–861)	0.02
**(2) Measurement 3 weeks after the third dose of BNT162b2**			
**Patients on hemodialysis (U/mL)**	19000 (11750–61000)	19000 (12000–59000)	0.85
**Healthcare workers (U/mL)**	20000 (16000–36500)	21000 (14000–36750)	0.68
**Increasing rate (2)/(1)**			
**Patients on hemodialysis**	126 (68–218)	91 (56–209)	0.19
**Healthcare workers**	45 (26–93)	35 (25–56)	0.02

Wilcoxon rank-sum tests were used.

Supplementary Table S3. The effect of diabetes on anti-spike immunoglobulin G antibody levels at the follow-up of the second dose.

	Diabetes	Non-Diabetes	*p*-Value
**(1) Measurement in October 2021**			
**Patients on hemodialysis (U/mL)**	220 (121–414)	202 (88–359)	0.14
**Healthcare workers (U/mL)**	564 (237–818)	609 (433–880)	0.63
**(2) Measurement 3 weeks after the third dose of BNT162b2**			
**Patients on hemodialysis (U/mL)**	21500 (12000–64000)	18000 (11000–57000)	0.20
**Healthcare workers (U/mL)**	19000 (16000–22000)	21000 (15000–36000)	0.58
**Increasing rate (2)/(1)**			
**Patients on hemodialysis**	124 (67–209)	121 (57–218)	0.90
**Healthcare workers**	39 (23–76)	35 (24–62)	0.93

Wilcoxon rank-sum tests were used.

Supplementary Table S4. Effect of glucocorticoids on anti-spike immunoglobulin G antibody levels at the follow-up of the second dose in patients undergoing hemodialysis.

	Glucocorticoid Users	Non-Users	*p*-Value
**(1) Measurement in October 2021**<br>**Patients on hemodialysis (U/mL)**	135<br>(61–187)	220<br>(104–393)	0.10
**(2) Measurement 3 weeks after the third dose of BNT162b2**<br>**Patients on hemodialysis (U/mL)**	23000<br>(13000–87250)	19000<br>(12000–61000)	0.63
**Increasing rate (2)/(1)**<br>**Patients on hemodialysis**	163<br>(81–1419)	122<br>(59–211)	0.22

Wilcoxon rank-sum tests were used.
